# Association between egoistic motivation and participation in sports volunteering among college students: the moderating effect of social norms and perceived organizational support

**DOI:** 10.3389/fpsyg.2025.1685476

**Published:** 2025-11-12

**Authors:** Dawei Zhang, Junying Wang, Shengguo Tian, Li Liu, Xiao Zhang

**Affiliations:** 1 School of P. E., Shanxi Normal University, Taiyuan, Shanxi, China; 2 School of Sports and Health Engineering, Hebei University of Engineering, Handan, Hebei, China; 3 School of Economics and Management, Shanxi Normal University, Taiyuan, Shanxi, China

**Keywords:** egoistic motivation, college students, sports volunteering, social norms, perceived organizational support

## Abstract

**Introduction:**

This study employs motivation theory as its primary analytical framework to examine the relationship between egoistic motivation and college students' participation in sports volunteer services, along with its underlying mechanisms. The research aims to provide a novel explanatory framework for understanding how egoistic motivation transforms into altruistic behavior and to offer practical implications for mobilizing college students' potential in sports volunteering.

**Methods:**

A structured questionnaire survey was conducted among 684 students (424 males and 260 females, aged 16–28) from six leading sports universities in China, including Shanghai University of Sport and Beijing Sport University. Data analysis was performed using STATA 16.0, utilizing logistic regression supplemented by tests of interaction and higher-order moderation effects to examine the roles of social norms and perceived organizational support.

**Results:**

The results revealed three key findings: (1) egoistic motivation had a significant positive effect on students' participation in sports volunteer service; (2) social norms significantly moderated the relationship between egoistic motivation and participation behavior; and (3) perceived organizational support further strengthened the moderating effect of social norms, demonstrating a significant higher-order moderation effect.

**Discussion:**

These findings provide new insights into the psychological mechanisms underlying the transformation from egoistic motivation to altruistic behavior. The study contributes to the development of more effective public fitness service systems by offering evidence-based approaches to enhance college students' engagement in sports volunteering activities.

## 1 Introduction

Sports volunteer service, as a typical form of public welfare, non-remunerated, and voluntary social activity, plays an irreplaceable role in the efficient operation of sports events and community sports activities worldwide. It not only compensates for shortages in human and managerial resources and improves service quality, but also fosters community cohesion and promotes the sustainable development of the sports sector (Michalska et al., 2025; Angosto et al., 2021; Adams and Deane, 2021). Within this system, sports volunteers represent an indispensable human resource. They make critical contributions not only to event organization, service delivery, and logistical support, but also to disseminating public welfare values, broadening civic participation, and shaping positive citizenship norms (Bang et al., 2019; Kim, 2018; Cuskelly and O'Brien, 2013). Consequently, the motivations underlying sports volunteer participation have become a central focus of scholarly inquiry. Existing research has sought to identify the conditions and drivers that shape individuals' decisions to engage in sports volunteering and to propose strategies that can enhance the sustainability of such participation (Bavaresco et al., 2022; Chutiphongdech et al., 2024; Han and Kim, 2025; Lorente et al., 2024; Rampasso et al., 2020).

A systematic review of the literature reveals that research on the motivations of sports volunteers has gradually shifted from early analyses focused on internal motivational components to broader explorations of external conditions such as educational demands, developmental benefits, and personal growth pathways (Rozmiarek, 2024; Chen et al., 2023, 2025; Kanar et al., 2025; Budreikaite et al., 2021; Whelan et al., 2023). This evolution reflects both the diversification of research agendas and the increasing concreteness of perspectives in the field. Theoretically, whether through the examination of internal motivational structures or the investigation of multi-factor interaction mechanisms, the analysis ultimately converges on two fundamental dimensions: egoistic motivation and altruistic motivation. For college students, who represent one of the most prominent participant groups in sports volunteering, empirical studies consistently demonstrate the joint influence of both motivational dimensions. Egoistic motives—such as enhancing professional skills, achieving personal recognition, and expanding social networks (Farrell et al., 1998; Zhang et al., 2020a)—intersect with altruistic motives—such as fulfilling social obligations, expressing selfless dedication, and promoting the development of sports initiatives (Wang and Gao, 2021; Ledford et al., 2018)—to shape students' participation behaviors. However, from the practical perspective of sustaining long-term sports volunteer engagement, existing studies remain insufficiently developed and under-theorized. As Rozmiarek et al. (2023) highlight, motivations for sustained involvement among college student volunteers have not received adequate scholarly attention, leaving important gaps in theoretical interpretation and practical application.

More specifically, the mechanisms through which egoistic and altruistic motivations influence college students' participation in sports volunteer service remain insufficiently understood. While the progression from “altruistic motivation” to “altruistic behavior” aligns with general cognitive logic, the transformation from “egoistic motivation” to “altruistic behavior” appears less intuitive and even counter-normative (Starshinova, 2019). Although some scholars have employed the concept of “altruism for self-interest” to interpret the ethical legitimacy of this transformation (Li and Xiao, 2011), the extent to which egoistic motivation functions as a triggering factor influencing students' volunteering behaviors—and the mechanisms through which it exerts such influence—remains largely underexamined. Addressing this gap constitutes an urgent direction for both empirical research and theoretical development.

Motivation theory posits that motivation is a critical factor influencing individuals' behavioral intentions and the occurrence of behavior (Petri and Govern, 2012). Based on this premise, research on college students' motivations to participate in sports volunteer service seeks to answer the questions of “why college students participate” and “how they can be motivated to sustain participation.” However, the relationship between motivation and behavior is more complex than is often assumed. Admittedly, motivation, as an important antecedent that triggers initial behavioral intentions, has been widely recognized for its guiding role at both the theoretical and empirical levels (Johnson et al., 2016; Thibaut, 2017; Penner, 2002). Nevertheless, the process through which behavior is generated is also influenced by external factors. For example, volunteer participation may stem entirely from an individual's internal willingness (Liu et al., 2015), or it may be shaped by social norms (Everett et al., 2015). Furthermore, situational factors such as perceived organizational support have been shown to facilitate the transformation of individuals' public service motivation into actual behavior (Dekel et al., 2022; Chen and Lin, 2016). Therefore, guided by motivation theory as the primary analytical framework, the present study introduces social expectations and perceived organizational support as moderating variables to examine the conditions under which egoistic motivation influences college students' participation in sports volunteer service.

## 2 Literature review and hypothesis generation

### 2.1 Relationship between egoistic motivation and participation in sports volunteer service

Research on the motivations for participating in sports volunteer service, both domestically and internationally, generally shares two common characteristics: first, college students are regarded as the primary target group of concern; and second, the focus is placed on explaining participation behaviors in the context of sports events. The overarching approach is to interpret the questions of “why participate” and “how to motivate” from the perspective of the participants themselves. Based on an empirical investigation of college students participating in volunteer service at the Beijing Winter Olympics, one study developed a five-dimensional motivation model that included skill enhancement, altruistic spirit, career development, social belonging, and reward-seeking. The results indicated that the “reward-seeking” dimension was the most significant factor influencing actual participation behavior (Zhou, 2021). Other studies have further pointed out that egoistic motivations, such as the pursuit of material rewards and self-value enhancement, are highly associated with college students' participation in event volunteer service (Wang, 2017; Hoye et al., 2008). In addition, research analyzing why students voluntarily engage in sports volunteer service has identified utilitarian purposes—such as fulfilling course requirements, expanding social networks, and gaining work experience—as primary drivers of early-stage participation (Hayton, 2016). Although previous studies have not explicitly employed the term “egoistic motivation” to describe college students' motivational preferences for sports volunteer service, various expressions of reward-seeking, self-improvement, and experience acquisition clearly reflect self-oriented rather than other-oriented tendencies. Therefore, the following hypothesis is proposed:

H1: Egoistic motivation has a significant positive effect on college students' participation in sports volunteer service.

### 2.2 The mediating role of social norms

Social norms encompass a social psychological phenomenon wherein the actions and opinions of others influence individual decision-making (Ouyang, 2022). These norms represent prevailing social opinions within social group behavior cognition, exert evaluative and regulatory effects on individual social behaviors (Osterhus, 1997; Shulman et al., 2017; Han and Zheng, 2016). According to normative focus theory, individuals engage in prosocial behavior not necessarily because of intrinsic moral awareness or altruistic purposes, but rather due to the influence of social norms (Cialdini et al., 1990). Existing research often classifies social norms into two dimensions—injunctive norms and descriptive norms—to reveal the effects generated by compulsory social pressure or unconscious behavioral guidance (Cialdini and Trost, 1998). As a specific manifestation of prosocial behavior, participation in sports volunteer service is likewise influenced by social norms. For example, expectations for sports volunteer participation expressed by governments and the general public toward college students, due to their identity characteristics, are generally higher than those for other demographic groups. Such socially constructed norms or moral expectations can help college students overcome the psychological limitations of self-interest, facilitating the connection between egoism and altruism, reconciling the tension between personal and societal interests (Li and Qin, 2006), and thereby increasing the likelihood of transforming egoistic motivation into altruistic behavior. Based on this reasoning, the following hypothesis is proposed:

H2: Social norms have a significant moderating effect on the relationship between egoistic motivation and college students' participation in sports volunteer service. Specifically, the higher the level of perceived social expectations, the stronger the positive association between egoistic motivation and participation behavior; conversely, the lower the perceived social expectations, the weaker the positive association.

### 2.3 Higher-order moderating role of perceived organizational support

Perceived organizational support (POS) refers to individuals' perception of the extent to which the organization values their contributions and cares about their wellbeing (Zhou and Jia, 2014), representing a subjective evaluation of their status within the organizational context (Fuller et al., 2006). Given that organizational affiliation is a defining feature distinguishing volunteer service from individual helping behavior (Snyder and Omoto, 2008), both domestic and international scholars have incorporated POS into existing research frameworks to examine its potential influence on volunteer participation. Prior studies have demonstrated that organizational support in terms of discretionary time and resources not only reduces the costs and constraints associated with volunteering but also enhances individuals' engagement in such activities (Booth et al., 2009). Furthermore, researchers have analyzed the moderating role of perceived organizational support between volunteers' willingness and their actual behavior from two perspectives—emotional support and instrumental support. Specifically, organizations can provide role clarity, systematic training, and logistical resources to meet individuals' basic needs, while simultaneously strengthening a sense of belonging through recognition, care, and team-building. Ultimately, these processes, operating within the dynamic balance of resource conservation and social exchange, have been shown to facilitate sustained volunteering behavior among organizational members (Wang et al., 2016; Kurtessis et al., 2017; Xu et al., 2020; Kao et al., 2023; Bang et al., 2023; Chiu et al., 2024).

Within the domain of sports volunteer service, as an extension of general volunteering, the role of perceived organizational support (POS) has likewise been empirically validated. Research indicates that motivation alone is insufficient to predict sports volunteers' willingness to engage in service, whereas POS emerges as a key predictor of their future participation behavior (Giel and Breuer, 2020). The underlying reason is that sports volunteer service is typically carried out in highly professionalized contexts, where volunteers are expected to possess a thorough understanding of complex event organization, rigorous role requirements, and standardized service protocols. Such knowledge and skills are most often acquired through the training and management provided by recruiting organizations or affiliated sports clubs (Won et al., 2024; Su et al., 2024; Ali et al., 2023). In addition, organizational systems that offer coordinated command structures and legal protections help reduce or even prevent disputes over rights and safety risks during service. These forms of institutional support not only safeguard volunteers but also facilitate the transformation of willingness into actual participation (Traeger et al., 2023; Mahmood and Pa, 2023; Bang et al., 2025).

At the same time, perceived organizational support can enhance the impact of informal networks and stable interpersonal relationships formed with the general public and other external actors. Specifically, behaviors shared within informal networks cannot be enforced through formal organizational mandates; instead, they rely on institutional and emotional support provided by the organization (Bock and Kim, 2002). From the perspective of organizational support, previous studies have developed theoretical models such as “informal networks–perceived organizational support–individual innovative behavior,” finding that perceived organizational support positively moderates the relationship between informal networks and individual innovation (Huang, 2014). Although few studies have examined sports volunteer service participation from this angle, it can be inferred that when college students not only perceive participation expectations from external groups such as the general public but also receive recognition, attention, and support from internal organizational members, their concerns about egoistic motivation are likely to be alleviated, thereby increasing the likelihood of participating in sports volunteer service. Based on this reasoning, the following hypothesis is proposed:

H3: Perceived organizational suppor significantly moderates the interaction effect of egoistic motivation and social norms on college students' participation in sports volunteer service. Specifically, in the low-perceived organizational suppor condition, the interaction effect of egoistic motivation and social norms on participation behavior is not significant; in the high-perceived organizational suppor condition, the interaction effect is significant.

**Figure 1:**
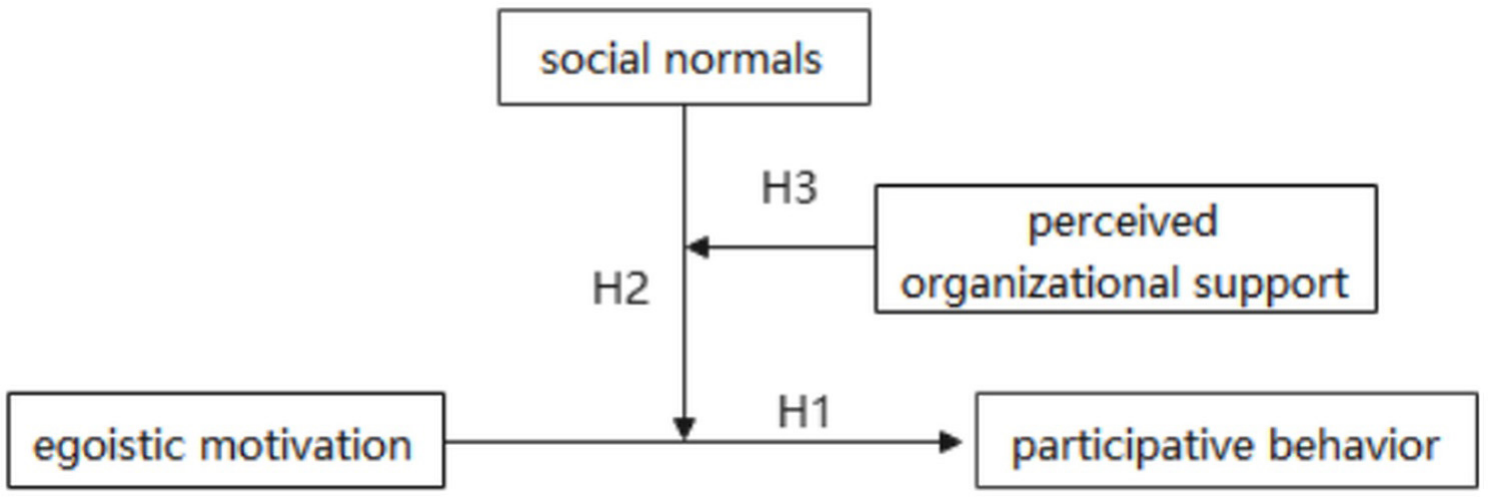
Hypothesized a mediation model.

In summary, the present study aims to examine the impact of egoistic motivation on college students' participation in sports volunteer service, as well as the moderating mechanisms of social norms and perceived organizational support (see Figure 1). Specifically, the relationship between egoistic motivation and college students' participation in sports volunteer service is moderated by social norms; furthermore, this moderating effect is subject to a higher-order moderation by perceived organizational support, such that perceived organizational support significantly influences the interaction between egoistic motivation and social norms in predicting participation behavior.

## 3 Materials and methods

### 3.1 Participants

To highlight the professionalized nature of sports volunteer service and to respond to the practical demand for professional volunteer service that emphasizes the knowledge and skills of professionals (McColl-Kennedy et al., 2015), this study selected students from several well-known sports-specialized universities in China—Shanghai University of Sport, Beijing Sport University, Wuhan Sports University, Nanjing Sport Institute, Chengdu Sport University, and Capital University of Physical Education and Sports—as survey participants. These institutions were chosen not only because they are located across eastern, central, and western regions of China, thereby ensuring spatial representativeness, but also because their students are more frequently engaged in sports volunteer service and thus possess relatively rich practical experience. This provided a reliable and representative data foundation for analyzing the relationship between motivation and behavior. The research protocol was reviewed and approved by the Science and Technology Ethics Committee of Shanxi Normal University. A snowball sampling approach was adopted because sports volunteers do not constitute a fixed population, making purely random sampling infeasible. The data collection procedure involved two steps: first, researchers identified active participants in sports volunteer service by visiting student associations and volunteer organizations on campus; second, these key participants were asked to distribute online questionnaires within their respective organizations to maximize sample coverage. Using this procedure, a total of 802 questionnaires were distributed, and 684 valid responses were obtained, yielding a valid response rate of 85.29%. In terms of demographic composition, 438 participants (64.04%) were aged 16–23 years, 224 (32.74%) were aged 24–27 years, and 22 (3.32%) were aged 28 years or older. Regarding gender, 260 participants (38.01%) were female and 424 (61.99%) were male. With respect to educational level, 448 participants (65.5%) were undergraduate students, and 236 (34.5%) were master's degree students. Regarding political affiliation, 142 participants (20.76%) were members of the Communist Party of China, 5 (0.73%) were members of democratic parties, 405 (59.21%) were members of the Communist Youth League, and 132 (19.3%) were unaffiliated with any political organization.

### 3.2 Measurement

All measurement instruments employed in this study were derived from well-established and psychometrically validated scales used in prior research. To ensure that the measurement items accurately reflected the context of college students' participation in sports volunteering, certain item wordings were contextually adapted. For instance, the term “event volunteering” was replaced with “sports volunteering,” and “employee or company” was replaced with “college student or school.” The adaptation process involved two steps: first, three experts from the fields of sports science, management, and psychology, along with two doctoral candidates, reviewed and revised the wording of the items to ensure suitability for the college student population. Second, a pilot test was conducted with 15 college students majoring in sports science to identify and remove any potentially ambiguous items, thereby improving the clarity and relevance of the questionnaire.

#### 3.2.1 Egoistic motivation scale

Egoistic motivation was measured using items adapted from the Volunteer Functions Inventory (VFI) developed by Clary et al. (1998) and the Olympic Volunteer Motivation Scale (OVMS) proposed by Giannoulakis et al. (2007), specifically focusing on the egoistic dimension. These items have been widely validated in previous empirical studies, demonstrating strong measurement validity. The final scale consisted of four items rated on a five-point Likert scale (1 = strongly disagree, 2 = disagree, 3 = neutral, 4 = agree, and 5 = strongly agree). Higher average scores indicated higher levels of egoistic motivation. The reliability of the egoistic motivation scale was assessed using Cronbach's α coefficient, which yielded a value of 0.934, indicating excellent internal consistency. To further evaluate construct validity, the Kaiser–Meyer–Olkin (KMO) test and Bartlett's test of sphericity were conducted. Results showed a KMO value of 0.854 and a significant Bartlett's test (χ^2^ = 2377.601, df = 6, *p* < 0.001), confirming that the scale demonstrated good validity.

#### 3.2.2 Social norms scale

Social norms were assessed based on the conceptual distinction between descriptive and injunctive norms proposed by Cialdini et al. (1990), and items were adapted from the Social Norms Scale developed by Sun et al. (2020). The final version included six items rated on a 5-point Likert scale (1 = “strongly disagree” to 5 = “strongly agree”). Higher average scores reflected stronger perceived social norms. The scale demonstrated high internal consistency (Cronbach's α = 0.921). Validity testing showed a KMO value of 0.913 and a significant Bartlett's test result (χ^2^ = 2966.560, df = 15, p < 0.001), affirming good construct validity.

#### 3.2.3 Perceived organizational support scale

Perceived organizational support was measured using a six-item scale adapted from the Perceived Organizational Support Scale developed by Eisenberger et al. (1986) and its culturally adapted version by Chen and Lin (2016). The items were rated on a 5-point Likert scale (1 = “strongly disagree” to 5 = “strongly agree”), with higher scores indicating stronger perceptions of organizational support. The scale showed excellent internal consistency (Cronbach's α = 0.935). Validity analysis produced a KMO value of 0.933 and a significant Bartlett's test result (χ^2^ = 4383.132, df = 15, *p* < 0.001), affirming good construct validity.

### 3.3 Statistical analysis

Under a standardized instructions, students who agreed to participate in the study were required to independently complete the questionnaire in a distraction-free environment to ensure the authenticity and validity of the data. Upon completion of data collection, the dataset was subjected to statistical processing and analysis using STATA 16.0. The analytical procedure consisted of the following steps: (1) testing for common method bias; (2) conducting descriptive statistical analysis; (3) examining the correlations among the variables; and (4) testing each research hypothesis sequentially to ensure the scientific rigor and robustness of the research findings.

## 4 Results

### 4.1 Common method biases test

This study employed a self-report method for data collection, which may introduce the risk of common method bias. To address this issue, Harman's single-factor test was conducted on all items measuring college student volunteer participation behavior. The results indicated that the variance explained by the first unrotated factor was 31.235%, which is below the critical threshold of 40%. The Kaiser–Meyer–Olkin (KMO) value was 0.967, and Bartlett's test of sphericity yielded a chi-square value of 12,454.961 (df = 190, *p* < 0.001). These results suggest that CMB was within an acceptable range and that the data were suitable for subsequent analyses.

### 4.2 Descriptive statistical analysis

In this study, the dependent variable was college students' participation in volunteer service, coded as 1 for participation and 0 for non-participation. The core independent variable was egoistic motivation, measured using four items such as “Participating in sports volunteer service can broaden my horizons and increase my knowledge.” The scores for these four items were summed to create a composite score, which was then averaged, with higher values indicating higher levels of egoistic motivation. Social norms served as the moderating variable and were measured using six items, including “My classmates often invite me to participate in sports volunteer service.” The scores for these six items were summed and averaged, with higher values reflecting stronger perceived social norms. Perceived organizational support was included as a higher-order moderating variable, assessed through six items such as “My school or organization strongly encourages participation in volunteer service.” The scores for these six items were summed and averaged, with higher values indicating greater perceived organizational support. As control variables, demographic variables including gender, age, education level, and political affiliation were incorporated into the analysis. Descriptive statistics for all variables are presented in Table 1.

**Table 1:** Descriptive statistics of different physical activity levels (*N* = 684).

**Variables**		**Define and assign values**	**M**	**SD**
Dependent variable	Participation behavior	Participate or not? 1 = yes, 0 = no	0.355	0.479
Independent variables	Egoistic motivation	Participating in sports volunteer service can broaden my horizons and increase my knowledge.	3.852	0.956
		Participating in sports volunteer service can enhance my overall quality and professional confidence.	3.95	0.95
		Participating in sports volunteer service can increase my job opportunities and competitiveness.	3.871	0.978
		Participating in sports volunteer service can provide appropriate remuneration or rewards.	3.81	0.958
	Social norms	The enthusiasm of classmates around me for participating in sports volunteer service is high.	3.623	1.019
		The enthusiasm of classmates around me for participating in sports volunteer service is high.	3.635	0.967
		Parents or friends believe i should participate in some sports volunteer service.	3.677	1.002
		Teachers or coaches will provide me with opportunities for sports volunteer service.	3.678	0.99
		Parents or friends believe i should participate in some sports volunteer service.	3.803	0.925
		I can sense the expectations of the public for us to participate in sports volunteer service.	3.833	0.993
	Perceived organizational support	The organization i belong to, such as my school or club, encourages me to participate in sports volunteer service.	3.743	0.984
		The organization i belong to, such as my school or club, frequently disseminates information about sports volunteer service.	3.724	0.961
		The organization i belong to, such as my school or club, conducts necessary service participation training.	3.675	0.969
		The organization i belong to, such as my school or club, has relatively fixed sports assistance recipients.	3.673	0.978
		The organization i belong to, such as my school or club, provides spiritual or material rewards during service participation.	3.709	0.969
		The organization I belong to, such as my school or club, offers assistance during service participation.	3.684	0.953
Control variables	Gender	1 = male, 0 = female	0.62	0.486
	Age	age	22.45	2.549
	Education	1 = undergraduate, 2 = Master's student	1.345	0.476
	Political status	1 = Communist party member, 2 = democratic party member, 3 = communist youth league member, 4 = general public	2.77	0.99

### 4.3 Correlation analysis

Pearson's correlation analysis was conducted to examine the relationships among all study variables, and the results are presented in Table 2. The findings indicate that all pairs of variables were significantly correlated, with correlation coefficients below 0.60. This suggests that although the relationships between the variables were statistically significant, they were not high enough to indicate problematic multicollinearity. In addition, the variance inflation factor values for all variables were below the threshold of 10, further confirming the absence of multicollinearity concerns. Therefore, the dataset was deemed suitable for regression analysis.

**Table 2:** Correlation analysis of variables.

**Variables**	**1**	**2**	**3**	**4**	**5**	**6**	**7**	**8**
Gender	1.000							
Age	0.044	1.000						
Education	0.129^**^	0.632^**^	1.000					
Political status	−0.005	0.133^**^	0.006	1.000				
Egoistic motivation	0.058	0.052	0.047	0.032	1.000			
Perceived organizational support	0.089^*^	0.102^**^	0.116^**^	0.063	0.412^**^	1.000		
Social norms	0.072	0.117^**^	0.100^**^	0.075	0.439^**^	0.502^**^	1.000	
participation behavior	0.015	0.063	0.121^**^	0.021	0.543^**^	0.528^**^	0.534^**^	1.000

^*^p < 0.1, ^**^p < 0.05.

### 4.4 Hypothesis testing

A hierarchical regression approach was employed to examine the effect of egoistic motivation on college students' participation in sports volunteer service, as well as the moderating roles of perceived organizational support and social norms. In Step 1, the control variables—gender, age, education level, and political affiliation—were entered into the model. Step 2 tested the main effects of egoistic motivation, perceived organizational support, and social norms on participation in sports volunteer service. In Step 3, the two-way interaction terms (egoistic motivation × perceived organizational support, egoistic motivation × social norms, and perceived organizational support × social norms) were added to examine the two-way interaction effects. In Step 4, the three-way interaction term (egoistic motivation × perceived organizational support × social norms) was included to test the higher-order moderating effect. The results of the regression models are reported in Table 3.

**Table 3:** Results of regression model testing.

**Variables**	**Model 1**	**Model 2**	**Model 3**	**Model 4**
Cons	0.211	−1.119^***^	−1.381^**^	−1.007^**^
Gender	−0.008	0.582^**^	0.561^**^	0.452^**^
Age	0.023	0.052	0.056	0.047
Education	−0.630^***^	−0.754^***^	−0.787^***^	−0.845^**^
Political status	0.052	0.002	−0.003	−0.019
Egoistic motivation		1.843^***^	0.335^**^	0.244^**^
Social norms		0.460	0.541^**^	0.177
Perceived organizational support		0.073^**^	0.058^**^	0.028^***^
Egoistic motivation^*^social norms			0.113^**^	0.044^*^
Egoistic motivation^*^perceived organizational support			1.545^***^	0.691^***^
Perceived organizational support^*^social norms			0.327	0.054^**^
Egoistic motivation^*^social norms^*^perceived organizational support				0.139^**^
LR chi^2^	10.82	327.94	335.16	341.38
Prob > chi^2^	0.028	0.000	0.000	0.000
Pseudo R^2^	0.012	0.368	0.377	0.383

^*^p < 0.1, ^**^p < 0.05, ^***^p < 0.01.

In terms of direct effects, Model 2 (Table 2) showed that the coefficient of egoistic motivation on college students' participation in sports volunteer service was 1.843, which was significant at the 0.01 level (*p* < 0.01). This indicates that egoistic motivation significantly promotes participation in sports volunteer service. The regression coefficient for social norms was 0.460, which did not reach statistical significance (*p* > 0.05), suggesting that the independent effect of social norms was not significant in this model. The coefficient for perceived organizational support was 0.073, which was significant at the 0.05 level (*p* < 0.05), indicating that higher levels of perceived organizational support significantly enhance participation levels. Therefore, Hypothesis H1 is supported.

**Figure 2:**
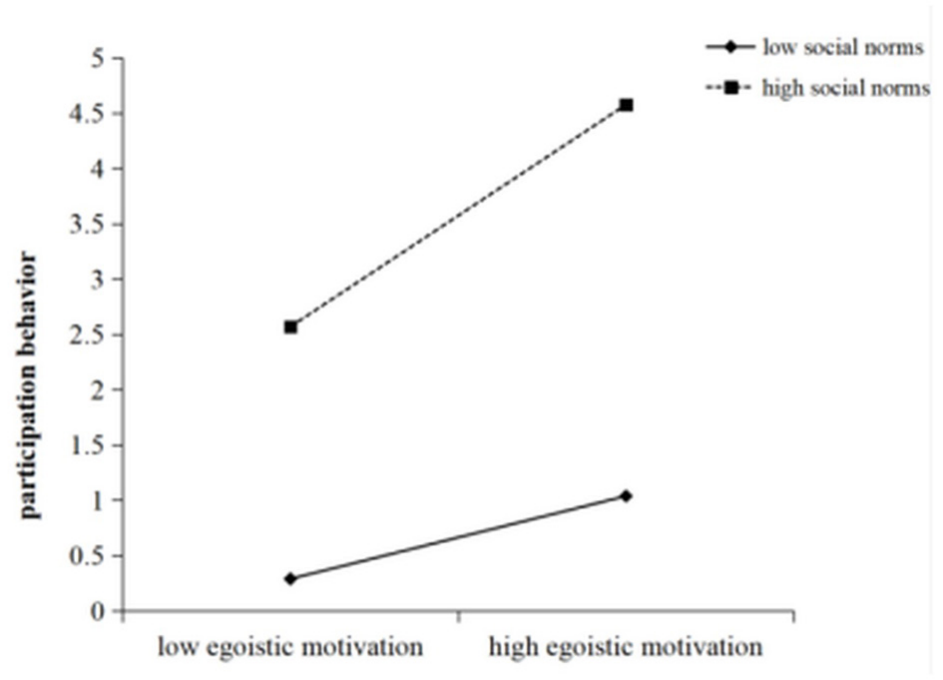
The interaction between egoistic motivation and social norms.

Regarding the two-way interaction effect, as shown in the regression results of Model 3 in Table 2, the interaction term between egoistic motivation and social norms had a coefficient of 0.113, which was significant at the 0.05 level (*p* < 0.05), indicating that social norms significantly moderate the relationship between egoistic motivation and participation in sports volunteer service. The interaction term between egoistic motivation and perceived organizational support had a coefficient of 1.545, significant at the 0.01 level (*p* < 0.01), suggesting that perceived organizational support also plays a significant moderating role. The interaction term between perceived organizational support and social norms had a coefficient of 0.327, which did not reach statistical significance (*p* > 0.05). Thus, Hypothesis H2 is supported. To further examine the moderating effect of social norms, participants were divided into a high social norms group and a low social norms group based on the mean ± 1 standard deviation, and an interaction plot was generated (see Figure 2). Simple slope analysis revealed that in the high social norms group, the effect of egoistic motivation on participation behavior was marginally significant (simple slope = 0.404, SE = 0.211, *t* = 1.918, *p* = 0.055), whereas in the low social norms group, the effect was not significant (simple slope = 0.206, SE = 0.149, *t* = 1.380, *p* = 0.168). These results indicate that higher levels of social norms strengthen the positive impact of egoistic motivation on sports volunteer service participation.

**Figure 3:**
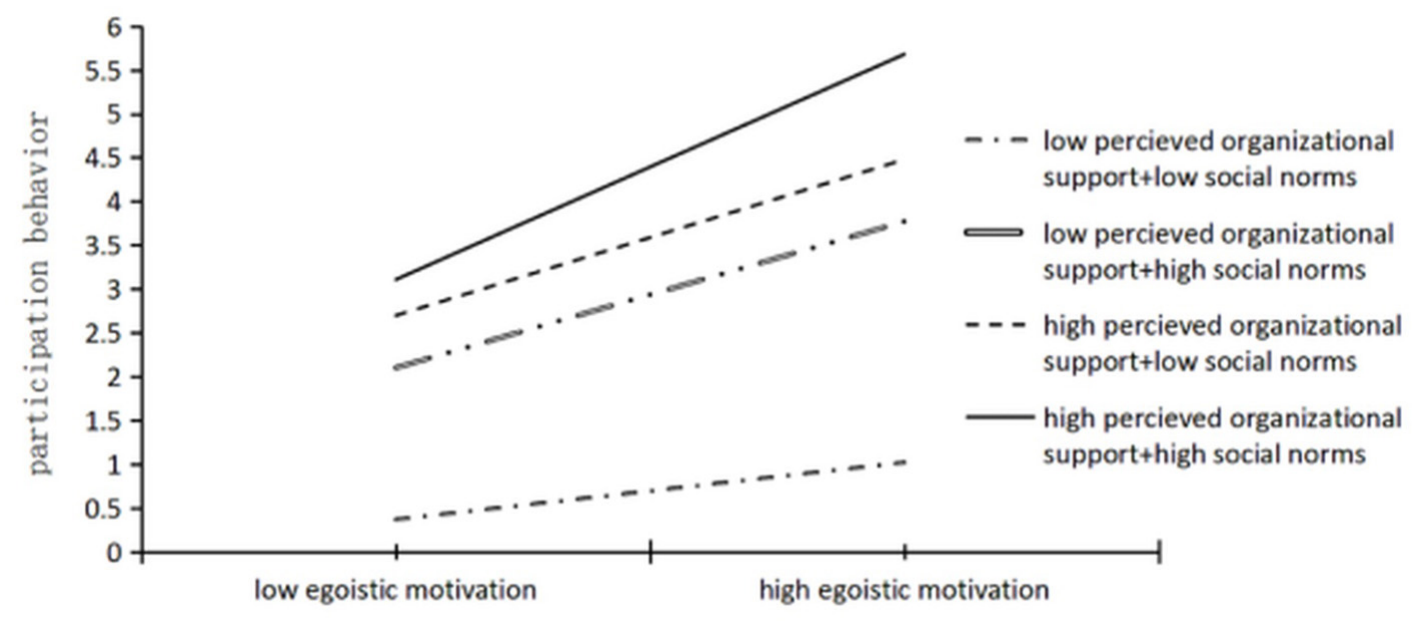
The three–order interaction of egoistic motivation, social norms and perceived organizational support.

Regarding higher-order moderation, the regression results of Model 4 in Table 2 indicate that the three-way interaction among egoistic motivation, perceived organizational support, and social norms had a coefficient of 0.139, which was significant at the 0.05 level (*p* < 0.05), thereby supporting Hypothesis H3. Further moderation analysis revealed that in the high perceived organizational support group, the interaction effect between egoistic motivation and social norms had a significant positive impact on college students' participation behavior (simple slope = 0.212, *F* = 7.928, *p* < 0.001), whereas in the low perceived organizational support group, this interaction effect was not significant (simple slope = −0.184, *F* = 0.165, *p* > 0.05). To provide a more nuanced understanding of the three-way interaction, social norms and perceived organizational support were each divided into high and low groups based on the mean ± 1 standard deviation, resulting in four scenario combinations (see Figure 3). Simple slope analyses were then conducted to decompose the higher-order moderation effect. Results showed that in the low perceived organizational support group, egoistic motivation had no significant effect on participation behavior for the low social norms group (simple slope = 0.008, SE = 0.027, *t* = 0.027, *p* > 0.05), but had a significant positive effect for the high social norms group (simple slope = 0.198, SE = 0.095, *t* = 2.080, *p* < 0.05). In the high perceived organizational support group, egoistic motivation had no significant effect on participation behavior for the low social norms group (simple slope = −0.016, SE = 0.050, *t* = 0.329, *p* > 0.05), but had a significant positive effect for the high social norms group (simple slope = 0.103, SE = 0.024, *t* = 4.290, *p* < 0.01).

Tests of slope differences across the four conditions in Figure 3 revealed significant differences between:Low social norms, low perceived organizational support vs. Low social norms, high perceived organizational support (*t* = −3.704, *p* < 0.001); High social norms, low perceived organizational support vs. High social norms, high perceived organizational support (*t* = 4.015, *p* < 0.001); Low social norms, low perceived organizational support vs. High social norms, low perceived organizational support (*t* = 3.803, *p* < 0.001); Low social norms, low perceived organizational support vs. High social norms, high perceived organizational support (*t* = 14.141, *p* < 0.001); Low social norms, high perceived organizational support vs. High social norms, high perceived organizational support (*t* = 4.415, *p* < 0.001). No significant difference was found between High social norms, low perceived organizational support and Low social norms, high perceived organizational support (*t* = 0.146, *p* = 0.884).

Regarding the effects of control variables, the regression results of Model 1 in Table 2 indicate that education level has a significant negative effect on college students' participation in sports volunteer service (β= −0.630, *p* < 0.01), suggesting that undergraduate students are more likely to participate in sports volunteer service compared to graduate students. In contrast, age, gender, and political affiliation show no significant influence on college students' participation in sports volunteer service.

## 5 Discussion

This study aimed to systematically examine the effect of egoistic motivation on college students' participation in sports volunteer service and to further identify the contextual conditions under which this effect occurs. To this end, social norms and perceived organizational support were incorporated into the analytical framework linking egoistic motivation and participation behavior, forming a higher-order moderated model. Empirical results supported all proposed hypotheses: (1) egoistic motivation was positively associated with sports volunteer service participation among college students; (2) social norms significantly moderated this relationship; and (3) social norms and perceived organizational support jointly exerted a significant three-way interaction effect on the relationship between egoistic motivation and participation behavior.

### 5.1 Relationship between egoistic motivation and college students' participation in sports volunteer service

The debate over whether volunteer participation is driven by altruistic or egoistic motives has been long-standing in academia. A growing body of empirical research has reached consensus that both motives jointly influence volunteer behavior (Jain, 2016). Studies have shown that the egoistic–altruistic motivational structure is stage-specific, with its balance shifting with age: young volunteers are more likely driven by egoistic factors, whereas with increasing age, their motivations tend to shift toward selfless altruism (Vorobieva and Skipor, 2021). Previous research focusing on college students' sports volunteer service has further differentiated the structure and dimensions of egoistic motivation (Huang and Wang, 2013). Although some scholars view the advocacy of voluntary social contribution and the pursuit of personal capital accumulation as contradictory in practice (Starshinova, 2019), the present study reaffirms that egoistic motivation positively influences students' engagement in sports volunteer activities. From the traditional perspective that egoistic and altruistic behaviors are opposing forces, their compatibility in shaping participation behavior necessarily depends on certain conditions. Accordingly, this study introduces social norms and perceived organizational support as moderators to examine the mechanism by which egoistic motivation may evolve toward altruistic behavior.

### 5.2 The moderating role of social norms

Empirical findings indicate that social norms significantly and positively moderate the relationship between egoistic motivation and sports volunteer service participation. This is consistent with prior theoretical assertions that a systematic, institutionalized social norm framework can effectively guide and strengthen individuals' propensity to engage in volunteer behavior (Li and Luo, 2019; Zhou, 2007; Tankard and Paluck, 2016; Legros and Cislaghi, 2020). Specifically, under high social norm conditions, egoistic motivation exerts a stronger positive effect on participation. The underlying logic is that as sports volunteer service becomes increasingly promoted and widespread in the sports domain, participation has become a broadly accepted social practice among college students (Zhang et al., 2020b). This trend increases students' —particularly sports majors perceived exposure to societal demand. Survey evidence suggests that such societal demand is conveyed via two primary channels: (1) top-down assignments from government agencies or sports organizations to universities, representing injunctive social norms, and (2) bottom-up initiatives by student associations to independently arrange volunteer activities, representing descriptive social norms. Together, these norms create an external environment that both encourages and legitimizes students' sports volunteer participation, allowing them to fulfill egoistic motives in a norm-consistent manner (Wu and Song, 2010; Miller and Prentice, 2016; Van der Linden, 2015).

### 5.3 The higher-order moderating role of perceived organizational support

Further analysis revealed that perceived organizational support significantly moderated the interaction between egoistic motivation and social norms in predicting participation behavior. Both simple slope analysis and slope difference tests demonstrated that the moderating effect of social norms varied with levels of perceived organizational support, thereby clarifying the conditions under which social norms exert their influence. Specifically, increased perceived organizational support tends to be accompanied by stronger social norms, as these norms encompass not only spontaneously formed social habits and moral codes (Wang, 2000; Tankard and Paluck, 2016) but also formal behavioral guidelines established by various organizations (Liu, 2021; Bicchieri, 2016; De Clerck et al., 2024). As educational institutions, universities influence students' sports volunteer participation by mobilizing, motivating, and rewarding them—actions that inherently reinforce social norms. Likewise, sports clubs and community sports organizations in which students voluntarily participate can heighten members' perception of social norms. Conversely, when universities or other informal organizations provide little support or attention to volunteer activities, students' normative perceptions must be shaped by external channels such as media campaigns or policy directives. Under conditions of low perceived organizational support and low social norms, egoistic motivation lacks sufficient activation factors. Thus, the positive influence of egoistic motivation on sports volunteer participation is most pronounced when both social norms and organizational support are high.

### 5.4 Practical implications

This study offers three key practical implications for promoting college students' participation in sports volunteer service: broadening motivational perspectives—In an era when sports volunteer service is becoming increasingly common, limiting discussions of participation solely to altruistic motives is overly narrow. While fostering altruism through educational guidance remains important, equal emphasis should be placed on designing mobilization, training, and incentive systems that appeal to egoistic motives from a win–win perspective, thereby enhancing actual participation. Leveraging social norms beyond compliance—The focus on social norms should extend beyond their rigid behavioral constraint function to their motivational role in converting egoistic motives into altruistic actions. This study indirectly addresses why college students, more so than other groups, are particularly suited for sports volunteer work—namely, the group-specific orientation of social norms. Strengthening organizational engagement—Organizationally structured action is a defining feature distinguishing volunteer service from informal helping behavior. By identifying and addressing the core needs of student participants, universities and affiliated organizations can develop more targeted support and incentive strategies. Furthermore, examining the psychological contract between students and their organizations may reveal how different organizational types or structures vary in their capacity to build social norms and stimulate participation, ultimately contributing to the sustainable development of sports volunteer organizations in higher education.

## 6 Conclusion

Based on the examination of egoistic motivation as a predictor of college students' participation in sports volunteer service and the conditions under which this effect occurs, the study yielded the following conclusions. First, egoistic motivation positively predicts participation behavior, reflecting a “self-serving altruism” tendency. Second, social norms significantly moderate the positive predictive effect of egoistic motivation on participation, such that the relationship is strengthened under higher levels of perceived social norms. Finally, perceived organizational support further moderates this interaction effect; specifically, the interaction between egoistic motivation and social norms significantly promotes participation only in contexts where organizational support is high. These findings offer valuable insights for stimulating college students' engagement in sports volunteer service across diverse cultural and institutional contexts by simultaneously strengthening social norms and organizational support.

## 7 Limitations and future directions

Although this study makes preliminary progress in uncovering the motivational mechanisms underlying college students' participation in sports volunteer service, several limitations should be acknowledged. First, the sample primarily consisted of students from sports universities or sports-related majors, in order to emphasize the professional attributes of sports volunteer service. However, field observations indicate that many volunteer activities in this domain involve non-specialized tasks—such as language translation, media promotion, and logistical support. Whether the present findings can be generalized to students without a sports-related academic background remains to be determined. Second, the present study conceptualized egoistic motivation as a single-dimensional construct, while existing literature has repeatedly highlighted the importance of altruistic motivation. The specific “role” altruistic motivation plays in the influence process of egoistic motivation—and the nature of their relationship—remains unexplored. Future research could address this gap by comparing the mechanisms and boundary conditions through which these two motives affect participation in sports volunteer service. Third, due to the constraints of measurement, participation in sports volunteer service was assessed as a binary outcome (“yes” or “no”). This approach does not capture differences in the degree of involvement. As a result, it remains unclear whether, and how, the influence of egoistic motivation varies across levels of participation. Future studies could adopt more fine-grained measures—such as total volunteer hours, frequency of participation, or task complexity—to explore whether the strength and direction of egoistic motivation's effects change according to the intensity or continuity of engagement. Fourth, due to the characteristics of the surveyed population, this study primarily employed a snowball sampling approach for questionnaire distribution. While this method was pragmatic, it may not fully capture the heterogeneity and diversity of sports volunteers, and the results are susceptible to the pace and psychological state of the respondents. Future research should address this limitation by expanding the sample size and incorporating more diverse volunteer populations to enhance the representativeness and generalizability of the findings.

## Data Availability

The raw data supporting the conclusions of this article will be made available by the authors, without undue reservation.
